# Socially induced plasticity of the posterior tuberculum and motor behavior in zebrafish (*Danio rerio*)

**DOI:** 10.1242/jeb.248148

**Published:** 2024-11-22

**Authors:** Faith K. Heagy, Katie N. Clements, Carrie L. Adams, Elena Blain, Fadi A. Issa

**Affiliations:** Department of Biology, East Carolina University, Greenville, NC 27858, USA

**Keywords:** Aggression, Dopamine, Diencephalon, Mauthner neuron, Social dominance, Zebrafish

## Abstract

Social dominance is prevalent throughout the animal kingdom. It facilitates the stabilization of social relationships and allows animals to divide resources according to social rank. Zebrafish form stable dominance relationships that consist of dominants and subordinates. Although social status-dependent differences in behavior must arise as a result of neural plasticity, mechanisms by which neural circuits are reconfigured to cope with social dominance are poorly described. Here, we describe how the posterior tuberculum nucleus (PTN), which integrates sensory social information to modulate spinal motor circuits, is morphologically and functionally influenced by social status. We combined non-invasive behavioral monitoring of motor activity (startle escape and swim) and histological approaches to investigate how social dominance affects the morphological structure, axosomatic synaptic connectivity and functional activity of the PTN in relation to changes in motor behavior. We show that dopaminergic cell number significantly increases in dominants compared with subordinates, while PTN synaptic interconnectivity, demonstrated with PSD-95 expression, is higher in subordinates than in dominants. Secondly, these socially induced morphological differences emerge after 1 week of dominance formation and correlate with differences in cellular activities illustrated with higher phosphor-S6 ribosomal protein expression in dominants compared with subordinates. Thirdly, these morphological differences are reversible as the social environment evolves and correlate with adaptations in startle escape and swim behaviors. Our results provide new insights into the neural bases of social behavior that may be applicable to other social species with similar structural and functional organization.

## Teaser

**Summary:** Behavioral monitoring of zebrafish motor activity and histological analysis provide new insights into how social dominance affects the morphological and functional organization of the posterior tubercular nucleus implicated in regulating spinal motor circuits.

## INTRODUCTION

Decision making is critical for animal survival. In social species, decision making is context dependent, driven, in part, by social cues as animals compete for dominance. During social interactions, neural networks integrate social information and drive spinal circuits to produce adaptive behavior. One common view is that neural circuits underlying reflexive behaviors such as those involved in escape or freezing are ‘hard wired’ and resistant to physiological plasticity (Manoli et al., 2006). Conversely, social behaviors involving learning and memory that change as the social environment evolves are mediated by flexible circuits that allow for more adaptive behavioral responses (Anderson et al., 2014; Stagkourakis et al., 2020; Yavas et al., 2019). However, it is likely that hard-wired and flexible neural circuits overlap or share common elements that maximize behavioral adaptations (Stagkourakis et al., 2020). Although, social status-dependent plasticity of neural circuits involved in reflexive and adaptive behaviors has been extensively investigated in various systems (Edwards and Kravitz, 1997; Jung et al., 2020; Müller et al., 2008; Whitaker et al., 2021), our knowledge of how social dominance influences the morphological and functional organization of brain circuits involved in motor control remains poorly understood.

Decision-making circuits implicated in social regulation have been extensively studied in several mammalian and teleost fish species (Filby et al., 2010; O'Connell and Hofmann, 2011, 2012b). Several brain regions that include the diencephalon, hypothalamus and telencephalon form neural nodes that play important roles in social aggression. Within these regions, an upregulation of specific genes is linked to aggression, including *slc6a3*, which codes for the dopamine active transporter (DAT) and dopamine type 2 receptor (*drd2*) (Chen et al., 2005). The elevated expression of *slc6a3* is of particular interest given the regulatory involvement of dopaminergic pathways in social anxiety, depression, motivation and aggression (Filby et al., 2010; Schwab-Reese et al., 2020). Given that it is well established that aggressive interactions have profound physiological effects on many animals (Chen et al., 2005; Filby et al., 2010; Schwab-Reese et al., 2020), we aimed to provide insight into how the cellular mechanisms that underlie changes in decision making for motor circuits are modified in a status-dependent manner.

Zebrafish (*Danio rerio*) have emerged as a suitable model organism to study the neurobiology of social behavior. Zebrafish shoal and communally engage in social agonistic interactions to form stable social dominance relationships (Oliveira et al., 2011; Miller et al., 2017; Clements et al., 2018, 2023). These agonistic interactions are driven by hormonal and neural regulation of brain nuclei involved in social regulation (O'Connell and Hofmann, 2012a,b). Specifically, the dopaminergic system is widely known for its neuromodulatory regulation of motivated behavior, aggression and regulation of motor activity (Clemens et al., 2006; Haehnel-Taguchi et al., 2018; Weitekamp et al., 2017). Of particular importance, the posterior tuberculum (PT), within the ventral diencephalon, forms a functional network that receives visual information from the optic tectum (Mu et al., 2012; Vernier and Wullimann, 2009; Yamamoto et al., 2017), and integrates and relays such information via ascending projections to the striatum and descending projections into the spinal cord (Ryczko et al., 2013). Within the PT, the posterior tuberculum anterior rostral (PTar) and posterior tuberculum anterior caudal (PTac) neural clusters project posteriorly into the mesencephalic region to directly modulate the startle escape and swim circuits, and indirectly modulate hindbrain glycinergic nuclei (Haehnel-Taguchi et al., 2018; Mu et al., 2012; Ryczko et al., 2013), the excitability of which is socially influenced (Orr et al., 2021; Clements et al., 2023). One distinguishing feature of PTar/PTac neurons is their large almond-shaped somata by comparison with other neighboring dopaminergic neurons (e.g. posterior tuberculum posterior group, PTp), which facilitates their identification. Collectively, the importance of visual communication during agonistic interactions along with the input/output structural organization of the PT suggests a propensity towards socially mediated plasticity as animals fight for social dominance. However, the effects of social dominance on the morphological and functional organization of the PT and downstream motor circuits remains unknown.

Previously, we have demonstrated that the startle and swim behaviors are socially regulated. After 2 weeks of continuous social interactions, dominant animals increase their swimming activity and become less likely to startle, while subordinates display the opposite behavior pattern by reducing their swimming activity and increasing their startle sensitivity (Miller et al., 2017). Although the cellular mechanisms underlying this status-dependent shift in motor activity remain unknown, these changes are likely driven, in part, by descending neuromodulatory input from the PTar/PTac cluster into the spinal cord to modulate startle and swim behaviors (Haehnel-Taguchi et al., 2018; Mu et al., 2012). This notion is supported by recent evidence showing that dopaminergic regulation of the startle escape and swim behaviors is socially driven to modulate the excitability of the startle escape and swim circuits in a status-dependent manner (Clements et al., 2023). This lends credence to the hypothesis that social dominance may affect the morphological and functional activity of the posterior tuberculum nucleus (PTN). Here, we tested this hypothesis by examining the time course during which adaptations in motor activity emerge and their relationship to PTN morphological and functional plasticity. We show that the number of DAT+ neurons in the PTar/PTac neural cluster increases in dominant animals compared with social subordinates, while synaptic connectivity is higher in subordinates compared with dominants. These socially induced morphological differences develop between 9 and 14 days post-dominance formation and correlate with status-dependent cellular functional activities in phosphor-S6 ribosomal protein (PS6) expression. PS6 detects ribosomal proteins that were recently phosphorylated, indicative of increased protein synthesis and enhanced cellular activity (Wayne et al., 2023). Finally, we show that these status-dependent differences are highly plastic and reversible if animals are provided with opportunities for social rise or induced into social fall.

## MATERIALS AND METHODS

### Animal care and housing.

A zebrafish transgenic line with specific green fluorescent protein expression in DAT neurons Tg(*dat*:GFP) was provided by Dr Timothy Erickson and maintained at the core aquatic animal facility in group-housed tanks consisting of 15–20 adult zebrafish of mixed sex. The facility was kept at 28°C under a 10 h dark/14 h light cycle. Fish were fed live brine shrimp and pellet food twice daily. All experiments were conducted according to guidelines approved by East Carolina University's Institutional Animal Care and Use Committee.

### Behavioral isolation, pairing and observation process

Although female zebrafish engage in agonistic interactions and form short-term dominance relationships (Teles and Oliveira, 2016), they undergo diurnal ovulation with the corresponding hormonal fluctuations that lead to unstable levels of aggressive activity and instability of dominance relationships. Given that the objective of our study was to examine the long-term consequences of stable social dominance on PTar/PTac morphological and functional activity, we limited the scope of the study only to adult male zebrafish. Adult male Tg(*dat*:GFP) zebrafish, ranging from 3 to 12 months old were isolated physically and visually from conspecifics for 1 week in 23×13×6 cm individual tanks, to minimize prior social experience. Following isolation, two male fish of equal size and age, and identical genetic background were paired continuously for 7, 9 or 14 days. Aggressive interactions were observed daily for 2–3 min at midday, and the number of attacks and retreats was quantified as described previously to ensure dominance stability (Miller et al., 2017). When paired, zebrafish form dominance relationships within an hour after pairing by engaging in ritualized agonistic interactions. Social dominance was determined daily for each pair based on the total number of aggressive attacks (biting and chasing) and defensive retreats. Fish with a high number of attacks were considered dominants while those that retreated from the attacks were considered subordinates. As dominance matures after day 1, the level of aggressive activities declines whereby only dominants attack while subordinate fish retreat. None of the pairs tested experienced social reversals during the first 2 weeks of interactions.

### Startle escape and swim behavioral testing

Using a non-invasive approach, the muscular electric field potentials during Mauthner cell (M-cell)-mediated C-start escape and swim behaviors were recorded as described in detail elsewhere (Issa et al., 2011; Miller et al., 2017; Clements et al., 2023). Muscular field potentials were amplified (×1000, AM-Systems, model 1700) and low-pass filtered at 300 Hz and high-pass filtered at 1 kHz before signals were digitized using a Digidata 1550 digitizer and stored on a personal computer (Axoscope software, Molecular Devices). After behavioral observations on days 7, 9 or 14, the fish were placed individually in a bath electrode chamber and allowed to acclimate for 30 min before testing. Following acclimation, 1 min of spontaneous swimming activity was recorded. To test escape response sensitivity, auditory pulses at increasing decibels between 0 and 100 dB re. 20 μPa were randomly delivered (1.5 min interstimulus interval, ISI) with 5 dB increments; 1 ms square sound pulses were generated using Audacity software. Stimuli amplitudes were randomized to prevent learning and desensitization effects. Each decibel was repeated 3 times. Startle responses that occurred within 15 ms or less of the auditory pulse were considered M-cell-mediated escape responses (Korn and Faber, 2005; Issa et al., 2011; Marsden and Granato, 2015). Escape probabilities were determined among all social groups with all values provided as means±s.e.m. Swim burst analysis using Clampfit software was completed by detecting bursts larger than 12 mV in total amplitude, and the duration of the burst needed to be in the range 50–200 ms to be considered a swim burst. Instantaneous frequencies and average number of bursts per minute were tabulated. All data were graphed using GraphPad Prism software.

### Reversal behavioral experiments

Males were isolated for 1 week then paired continuously for 14 days. On day 14, two dominants from separate pairs were size matched to form a new (dominant versus dominant) pair. Similarly, subordinates from separate pairs were crossed to form new (subordinate versus subordinate) pairs. The new pairs were kept together continuously for an additional 14 days to allow formation of new dominance relationships (total 28 days of pairing). Isolation, pairing and behavioral testing of startle sensitivity and swimming activity was carried out as described earlier.

### Brain tissue extraction, preparation and slicing

Immediately following behavioral experiments, animals were euthanized in 0.2% MS-222, then the brains were extracted and placed in 4% paraformaldehyde overnight at 4°C. The following day, brains were washed repeatedly for 25 min in 1% phosphate buffered saline (PBS). Brain tissue were incubated overnight in 30% sucrose at 4°C for cryoprotection. Brains were embedded in OCT filled molds, oriented sagittally, then flash frozen in liquid nitrogen. Embedded brain tissues were sliced at 30 µm using a cryostat (Microm HM 550) and slices were placed onto Superfrost Plus Microscope slides (Fisher Scientific). Brain slices were allowed to dry completely onto the slides in dark containers for 10 min prior to staining.

### Immunohistochemistry

Post-synaptic density-95 (PSD-95) (Abcam #Ab18258) and phospho-S6 ribosomal protein (Ser235/236) antibody (Cell Signaling #2211) for PS6 staining were used, as previously tested and confirmed in zebrafish (Brenet et al., 2019; He et al., 2021). Slices were washed 3–4 times for 5 min each in 1% PBS then placed in 5% bovine serum and 2% goat serum blocking buffer (1–2 h) following cryo-sectioning. Another series of PBS washes (3–4 times for 5 min) was performed, and slices were stained with a rabbit polyclonal anti-PSD-95 primary antibody at a concentration of 1:200. In PS6 experiments, the tissue was incubated at a concentration of 1:600. Slides were then incubated overnight at 4°C. Following primary antibody incubation, an additional 3–4 PBS washes were performed, and slices were incubated for 1–2 h at room temperature in goat anti-rabbit Alexa Fluor-555 secondary antibody (Invitrogen #A21428) diluted at a 1:500 concentration. Unbound secondary antibody was removed by washing in PBS (3–5 times), then slides were mounted with one drop of Antifade gold reagent protectant (Invitrogen #P36980). Slides were cured in the fridge for 24 h then sealed.

### Imaging acquisition and analysis

Confocal images were obtained using a Carl Zeiss LSM 800 confocal microscope equipped with 40× oil objective lens. To standardize acquisition across animal groups, acquisition mode settings were kept constant across conditions. Once imaging was completed, confocal stacks were imported and analyzed using Imaris software (Oxford Instruments, v.9.3) for automated cell count, colocalization, fluorescence intensity and volumetric analysis. Cell count of PTar, PTac, PTp, lateral recess and paraventricular organ posterior part B (PVOpB) neurons was conducted by selecting either the spots or cell surface functions. All image analysis was conducted blind and cross-checked by multiple individuals. Fluorescence intensity analysis was conducted in ImageJ. Fluorescence intensity of PTar/PTac cells was measured by calculating the corrected total cell fluorescence (CTCF) according to the following equation: CTCF=integrated density−(cell area×background average fluorescence). CTCF of a minimum of 10 cells per brain slice was measured. PS6-expressing cells with either CTCF <6000 (low signal to noise threshold) or >800,000 (saturation of fluorescence) were excluded from analysis. Analyzed data were exported and tabulated into Excel and graphed in Prism (GraphPad, v.3) software.

### Statistical analysis

Prism was used to generate all graphs and statistical results. Statistical analysis was performed using one-way ANOVA test with Kruskal–Wallis multiple comparison *post hoc* test, two-tailed; data met the requirement of normal distribution, and no data points were excluded from analysis for any of the results presented. To compare M-cell startle escape response probability among the social groups, the data were curve fitted with a Boltzmann Sigmoidal curve fit with a non-linear regression. One-way ANOVA tests were performed to compare the average number of swim bursts per minute, and the number of PTac and PTar neurons at day 7, 9 and 14 of social pairing for each phenotype. A paired *t*-test was performed on somata volumetric differences between PTar, PTac and PTp cells as well as comparing cell number across experimental conditions relative to communal fish (controls). Control fish were males selected from tanks in which 15–20 fish of mixed sex were group housed. The selected fish were siblings of the experimental group fish. The controls were of similar size, age and genetic background to the experimental group fish. Chi-square analysis was performed on PSD-95 expression differences among social phenotypes. Statistical significance level was set to *P*<0.05. The R statistical environment was used to conduct principal component analysis (PCA) using the *prcomp*() function (R v.4.3.3, 2024; RStudio version 2023.12.1+402; https://www.R-project.org/). We used packages ggfortify (Tang et al., 2016), factoextra (https://CRAN.R-project.org/package=factoextra) and ggplot2 (Wickham, 2016) to visualize the results. The PCA was used to examine and visualize clustering of individuals according to social status groups based on the set physiological and behavioral variables measured. Each sample is represented as a point (or projection) and arrows represent the correlations of each variable to the principal components.

## RESULTS

### Sensitivity of the M-cell-mediated startle response is socially regulated

**Fig. 1.**
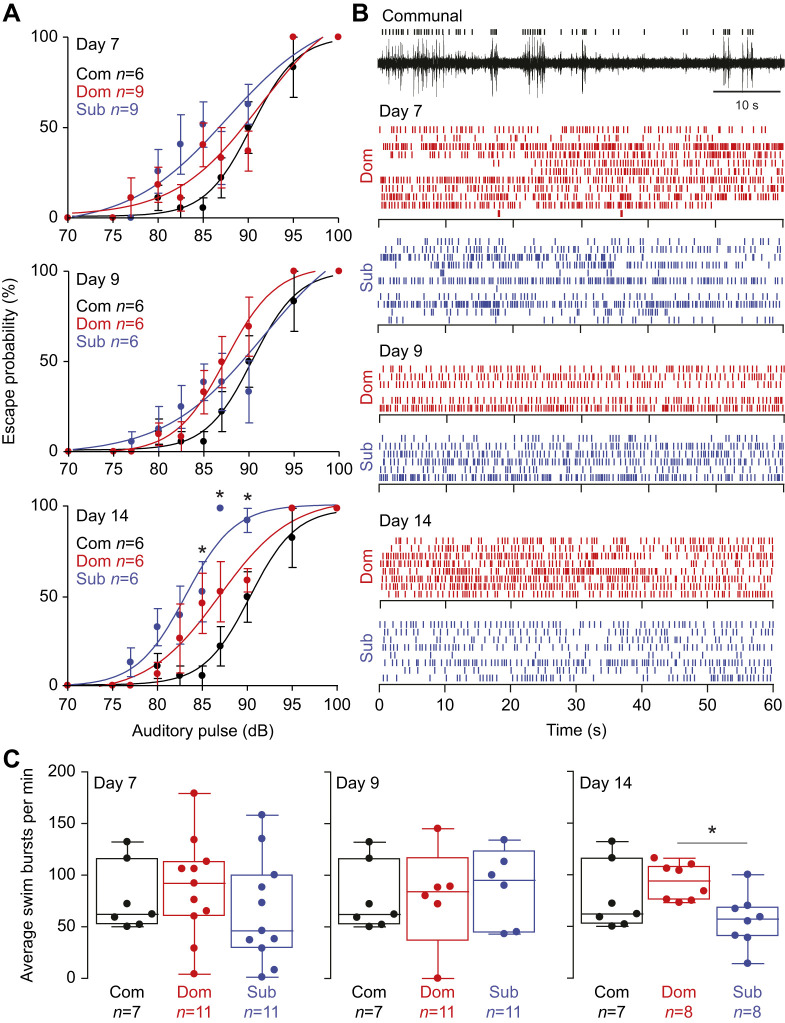
**Effect of social status on startle response and swim behaviors.** (A,B) Startle response probabilities of communal, dominant and subordinate (Com, Dom, Sub) zebrafish on days 7, 9 and 14 of social pairing (A) along with corresponding raster plots of swim burst activity in dominants and subordinates (B; communal control data not shown, refer to C for a summary of communal data). Each row in B represents the activity of an individual fish, and each vertical dashed line represents one swim burst (top trace is a representation of a raw far-field potential recording from a communal fish). (C) Summary box plots of the average swim bursts per minute among all three social groups (box denotes 95% of data, horizontal line within the box denotes the median, and error bars denote minimum and maximum values). Circles represent average individual fish spontaneous swim activity during a 1 min recording. One-way ANOVA with Kruskal–Wallis multiple comparison *post hoc* test (*P<0.05).

Prior work demonstrated that after 14 days of interactions, patterns of motor activity shift according to social rank, whereby dominants increase their swimming activity and reduce their startle sensitivity, while subordinates reduce their swimming and increase their startle sensitivity (Miller et al., 2017). However, the time course during which these behavioral adaptations emerge and their relationship to changes in brain morphology have not been examined. We hypothesized that during the early periods of dominance formation (days 7 and 9) there would be no social status-dependent differences as social relationships have not yet matured. To test this hypothesis, we measured the animals' startle escape sensitivity and swim behavior in dominants and subordinates at 7, 9 and 14 days of social interactions (Fig. 1). We found that the sensitivity of startle escape among all groups on day 7 and day 9 showed no significant differences (Fig. 1A; day 7: communals *n*=6, dominants *n*=9, subordinates *n*=9; day 9: communals *n*=6, dominants *n*=6, subordinates *n*=6) though there was a diverging trend in startle response where subordinates were slightly more sensitive and dominants were not as responsive. Those differences became progressively more pronounced and statistically different by day 14 [one-way ANOVA, Kruskal–Wallis *post hoc* test, communals versus subordinates (85 dB) *P*=0.0434, communals versus dominants (87 dB) *P*=0.053, dominants versus subordinates (90 dB) *P*=0.0226; communals *n*=6, dominants *n*=6, subordinates *n*=6].

As with the startle escape response, when we measured swimming activity among the three social groups, we found a corresponding change in the swimming pattern that emerged after 9 days and became significant by day 14 post-pairing (Fig. 1B). We found that dominants and subordinates did not vary in spontaneous swim activity on days 7 and 9, but by day 14, subordinates significantly decreased their swimming activity (Fig. 1C; one-way ANOVA, Kruskal–Wallis *post hoc* test; day 7: *P*=0.440, communals *n*=7, dominants *n*=11, subordinates *n*=11; day 9: *P*=0.8461, communals *n*=7, dominants *n*=6, subordinates *n*=6; day 14: *P*=0.0142, communals *n*=7, dominants *n*=8, subordinates *n*=8). These results show that although social dominance may be established quickly within the first week of social interactions, social status-dependent differences in motor activity emerge later between 9 and 14 days as dominance matures.

### Social status affects PT cell number

**Fig. 2.**
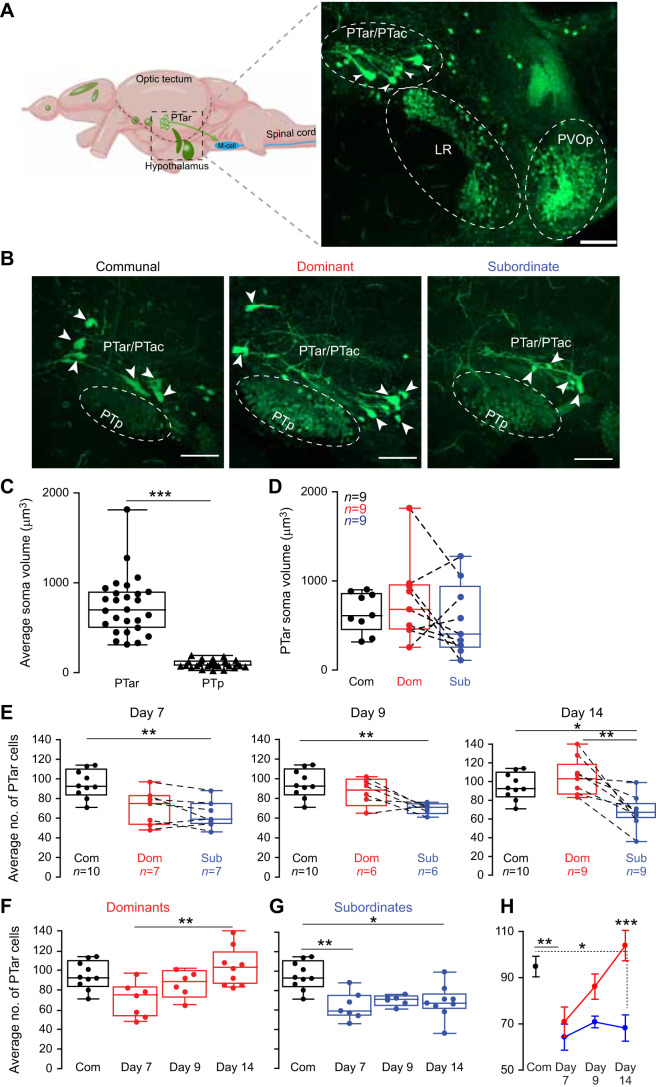
**Effect of social status on posterior tuberculum (PT) cell number.** (A) Schematic sagittal view of zebrafish approximate dopamine nuclei and a confocal projection of hypothalamic dopamine nuclei including the anterior rostral/anterior caudal PT (PTar/PTac) cluster, lateral recess (LR) and paraventricular organ posterior part B (PVOp; 30 µm thickness) in relation to the Mauthner neuron (M-cell). (B) Representative confocal projections from a communal, dominant and subordinate animal highlighting the PTar/PTac somata (arrowheads) and posterior PT (PTp) dopamine active transporter (DAT)-expressing cells (ovals) at 14 days post-pairing (scale bars: 30 µm). (C) Average soma volume for PTar/PTac and PTp cells in communal animals. (D) Comparison of PTar soma volume across social groups. Dashed lines denote pairwise comparison of each dominant with its subordinate counterpart. (E) Average number of PTar cells on days 7, 9 and 14. (F–H) Same data as in E but comparing time course changes in the average number of cells separately for dominants and subordinates relative to communals. For box plots in C–F, the box denotes 95% of data, the horizontal line within the box denotes the median, and error bars denote minimum and maximum values. C: *t*-test; D–H: one-way ANOVA with Kruskal–Wallis multiple comparison *post hoc* test (*P<0.05, **P<0.01, ***P<0.001).

The PT dopaminergic nucleus integrates sensory social information and relays those signals to spinal cord circuits including the M-cell startle escape circuit. Given the observed differences in escape sensitivity between dominants and subordinates, we hypothesized that the PTar/PTac cluster within the PT is prone to socially induced plasticity. First, we quantified the total number of PT cells in socially dominant, subordinate and communal controls between days 7 and 14 post-pairing (Fig. 2). As noted, the PT consists of several dopamine-expressing nuclei, and only the PTar/PTac cluster has large and almond-shaped cells that project and innervate spinal cord targets. To ensure accurate identification of the PTar/Ptac cluster, we measured and compared their soma volume to those of neighboring PT nuclei (e.g. PTp) (Fig. 2B,C). We found that the average soma volume of PTar/PTac clusters was significantly larger than that of the PTp nucleus, consistent with a prior report (Fig. 2C; *t*-test, *P*<0.0001, communal PTar/PTac *n*=27, PTp *n*=28) (Haehnel-Taguchi et al., 2018). But we found no differences in PTar/PTac soma volume among the three social phenotypes, nor was there a correlation in soma volume between dominant and subordinate pairs (Fig. 2D, one-way ANOVA, Kruskal–Wallis *post hoc* test, *P*=0.5008; communals *n*=9, dominants *n*=9, subordinates *n*=9). Next, we compared the number of PTar/PTac cells on days 7, 9 and 14 among the three social phenotypes. On days 7 and 9, communals had a significantly higher number of cells compared with subordinates, but there were no differences between communals versus dominants and dominants versus subordinates (Fig. 2E, one-way ANOVA, Kruskal–Wallis *post hoc* test, day 7: *P*=0.0036, communals *n*=10, dominants *n*=7, subordinates *n*=7; day 9: *P*=0.0088, communals *n*=10, dominants *n*=6, subordinates *n*=6). However, by day 14, the difference in the number of cells between dominants and subordinates became statistically significant (Fig. 2E; Movie 1; one-way ANOVA, Kruskal–Wallis *post hoc* test, *P*=0.0013, communals *n*=10, dominants *n*=9, subordinates *n*=9). Interestingly, in dominant animals, the average number of PTar/PTac neurons gradually increased over time and became significantly higher by day 14 compared with day 7 (Fig. 2F; one-way ANOVA, Kruskal–Wallis *post hoc* test, *P*=0.0116, communals *n*=10, dominants day 7 *n*=7, day 9 *n*=6, day 14 *n*=9). In subordinates, the number of cells remained consistently lower relative to that of communals (Fig. 2G; one-way ANOVA, Kruskal–Wallis *post hoc* test, *P*=0.0015, subordinates day 7 *n*=7, day 9 *n*=6, day 14 *n*=9). These results show that the PTN is prone to social plasticity.

To determine whether the effects of social dominance on dopaminergic nuclei is specific to the PTar/PTac or extends to proximate dopaminergic nuclei, we examined the lateral recess and PVOp nuclei that are adjacent to the PT (see Fig. 2A). The lateral recess and PVOp axonal projections are local and are thought to be involved in hormonal homeostatic regulation; thus, they are unlikely to be affected by social dominance (Haehnel-Taguchi et al., 2018). To test this hypothesis, we quantified the number of DAT-positive cells in the lateral recess and PVOp nuclei (Table 1). We found no statistical differences among all social groups in the PVOp and lateral recess regions (Table 1). This result suggests that the effect of social dominance on the PT is likely specific to dopaminergic nuclei that are involved in social regulation.

**Table 1.**
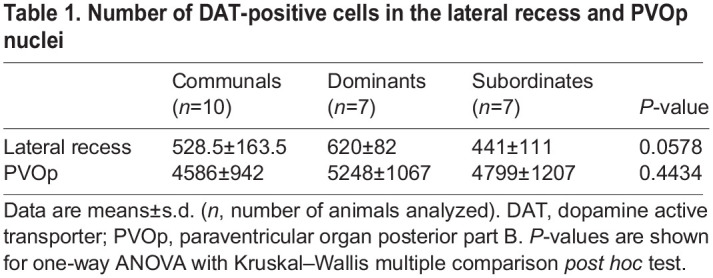
Number of DAT-positive cells in the lateral recess and PVOp nuclei

### Differences in motor activity and plasticity of PT cell number are not innate

**Fig. 3.**
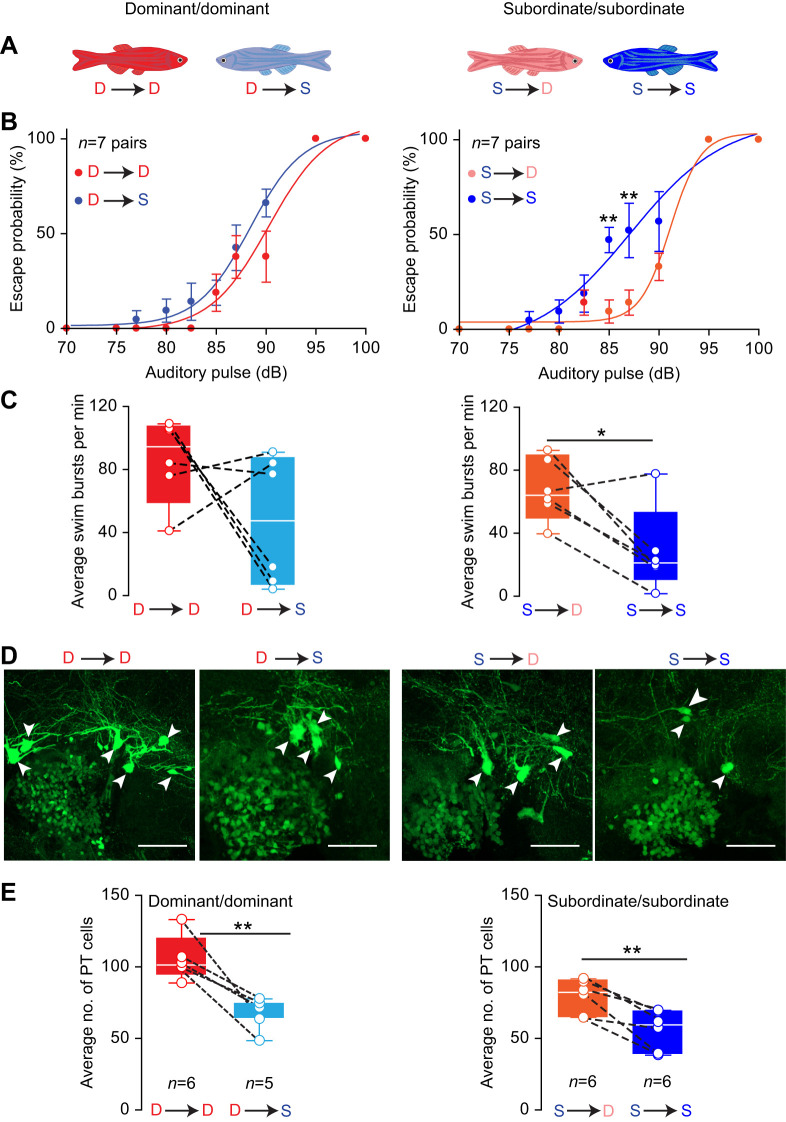
**Effect of social reversal on startle sensitivity, swim activity and PT cell number.** (A) Schematic illustration of experimental crossings (D, dominant; S, subordinate; where D→D remain dominant, D→S are descending subordinates, S→D are ascending dominants and S→S remain subordinate; refer to Materials and Methods for details). (B) Startle escape probability in dominant/dominant pairs (left) and subordinate/subordinate pairs (right). (C) Average number of swim bursts per minute in dominant/dominant pairs (left) and subordinate/subordinate pairs (right). (D) Representative confocal projections of dominants and subordinates from the dominant/dominant pairs and subordinate/subordinate pairs. Arrowheads indicate PTar cells (scale bars: 30 µm). (E) Average number of PTar/PTac cells from the respective animal crossings. For box plots in C and E, the box denotes 95% of data, the horizontal line within the box denotes the median, and error bars denote minimum and maximum values. Dashed lines denote pair-wise comparison of each dominant to its subordinate counterpart. B,C,E: two-tailed paired *t*-test (*P<0.05, **P<0.01).

The gradual divergence in PT cell number during the 2 weeks of social interactions suggested that social factors rather than innate differences underly the observed results (Fig. 2E–H). To investigate the robustness of social influence on PT morphology, we hypothesized that reversal in social conditions would correlate with reversal in motor behavior and PTN cell number. We tested this hypothesis by pairing dominants together to form new pairs (dominant versus dominant) and subordinates together to form new pairs (subordinate versus subordinate) and allowed the new pairs to establish social relationships for an additional 14 days of interactions (Fig. 3A). Under this scenario, in the dominant/dominant pairs, one of the animals would remain dominant while its partner would become a subordinate. Conversely, in the subordinate/subordinate pairs, one of the animals would become dominant while its counterpart would remain a subordinate. After 2 weeks of social interactions, we measured the animals' startle escape sensitivity, swimming activity and number of PTar/PTac cells (Fig. 3B–E).

In the dominant/dominant pairs, the sensitivity of the startle response of the descending subordinates (D→S) did not shift significantly compared with that of their dominant counterparts (D→D) (Fig. 3B). However, sensitivity of the startle response differed significantly between dominants and subordinates in the subordinate/subordinate pairs. The sensitivity of the startle response of the ascending dominants (S→D) declined significantly compared with that of their subordinate counterparts (S→S) (Fig. 3B; two-tailed paired *t*-test, 85 dB: *P*=0.0313, *t*=4.382, d.f.=6; 87 dB: *P*=0.0049, *t*=4.336, d.f.=6; *n*=7 pairs). Interestingly, the dominant/dominant pairs displayed higher aggressive activity and mortality rate (5 pairs were excluded from analysis as a result of mortality) compared with the subordinate/subordinate pairs.

Next, we measured spontaneous swimming activity. In the dominant/dominant pairs, swimming frequency of dominants that lost their ranks and became subordinates (D→S) declined compared with that of their dominant counterparts (D→D), but the decline was not statistically significant (Fig. 3C; two-tailed paired *t*-test, *P*=0.1910, *t*=1.512, d.f.=5; *n*=6 pairs). However, in the subordinate/subordinate pairs, swimming frequency of subordinates that ascended and became dominants (S→D) increased significantly compared with that of their subordinate counterparts (S→S) (Fig. 3C; two-tailed paired *t*-test, *P*=0.0192; *t*=3.403 d.f.=5, *n*=6 pairs). Interestingly, the increase in swimming frequency among the dominants (S→D) in the subordinate/subordinate pairs rose nearly to the level of those of dominants in the dominant/dominant pairs (D→D) and became statistically indistinguishable (Fig. 3C; two-tailed unpaired *t*-test, *P*=0.187, *t*=1.417, d.f.=10; *n*=6 animals). Finally, comparison of the swimming frequency between dominants in the dominant/dominant pairs relative to the subordinates in the subordinate/subordinate pairs showed significant differences (Fig. 3C; two-tailed unpaired *t*-test, *P*=0.003, *t*=3.886, d.f.=10; *n*=6 animals), but there were no significant differences between the subordinates in the dominant/dominant pairs compared with the subordinates in the subordinate/subordinate pairs (Fig. 3C; two-tailed unpaired *t*-test, *P*=0.3665, *t*=0.9459, d.f.=10; *n*=6 animals).

Finally, we examined the effect of social reversal on the number of PTar/PTac cells. In the dominant/dominant pairs, the number of PTar/PTac cells in dominants that lost their rank and became subordinates (D→S) declined significantly compared with that of their dominant counterparts (D→D) (Fig. 3E; two-tailed paired *t*-test, *P*=0.0079; dominants *n*=6, subordinates *n*=5). In the subordinate/subordinate pairs, we found that the number of PTar/PTac cells in dominants that ascended from submissive status (S→D) increased significantly compared with that of their subordinate counterparts (S→S) (Fig. 3E; two-tailed paired *t*-test, *P*=0.0057; dominants *n*=6, subordinates *n*=6). Collectively, the results show that the PTN and motor activity are socially regulated and highly plastic as animals rise and fall in dominance.

### Social status affects synaptic connection of the PT cells

**Fig. 4.**
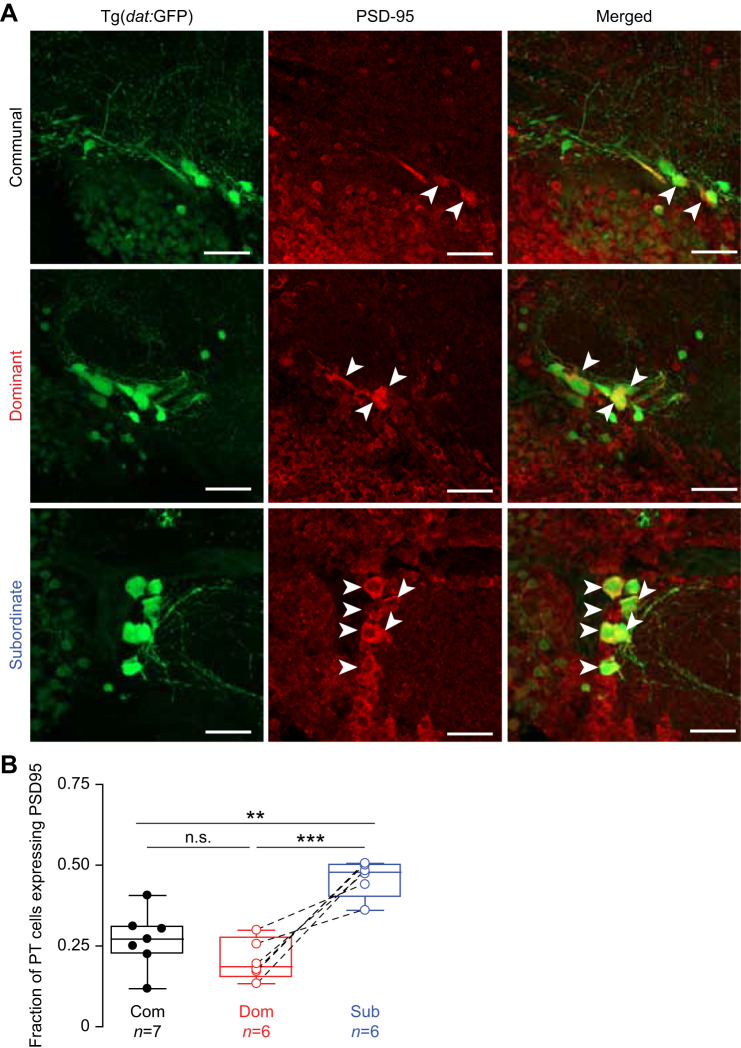
**Effect of social status on synaptic connection in the PTar nucleus.** (A) Representative confocal projections of PTar/PTac stained with PSD-95 in Tg(*dat*:GFP) communal, dominant and subordinate fish (scale bars: 30 µm). Arrowheads denote PTar/PTac cells expressing PSD-95. (B) Box plot of the fraction of PTar/PTac (PT) cells with axosomatic PSD-95 expression (day 14; box denotes 95% of data, horizontal line within the box denotes the median, and error bars denote minimum and maximum values). Dashed lines denote pair-wise comparison of each fish with its counterpart. Chi-square test (*P<0.01, **P<0.001).

The PT nucleus forms a functional network with extensive synaptic connectivity among its neurons. Cellular gain or loss is likely to impact its structural and functional organization. However, homeostatic mechanisms may play a significant role ensuring continued network functionality. We hypothesized that the observed differences in PTN cell numbers may lead to compensatory axosomatic connectivity to maintain PT functional integrity. We tested this hypothesis by staining for PSD-95 synaptic marker (Fig. 4; Movie 2). A ratio for each animal was computed by taking the total number of PTar/PTac cells that express PSD-95 and dividing by the total number of PTar/PTac cells. Using the average ratios of each social group, a Chi-square analysis was performed comparing all social phenotypes. Surprisingly, on average, 21±0.061% (mean±s.d., *n*=6) of PT neurons expressed PSD-95 in dominants, which is significantly lower compared with subordinates, which averaged 46±0.0542% (*n*=6) (Chi-square:14.0276, *P*=0.0002) (Fig. 4B). Average PT PSD-95 expression in communals was 27±0.0957% (*n*=7) and was not statistically different from that in dominants but was significantly different from that in subordinates (communals–dominants comparison: Chi-square: 0.9868, *P*=0.3205; communals–subordinates comparison: Chi-square: 7.7877, *P*=0.0053) (Fig. 4B). Overall, the results show that despite a decline in overall PTar/PTac cell number, subordinates have more PT cells that express PSD-95 compared with communals and dominants.

### Social status-dependent differences in PS6 expression

**Fig. 5.**
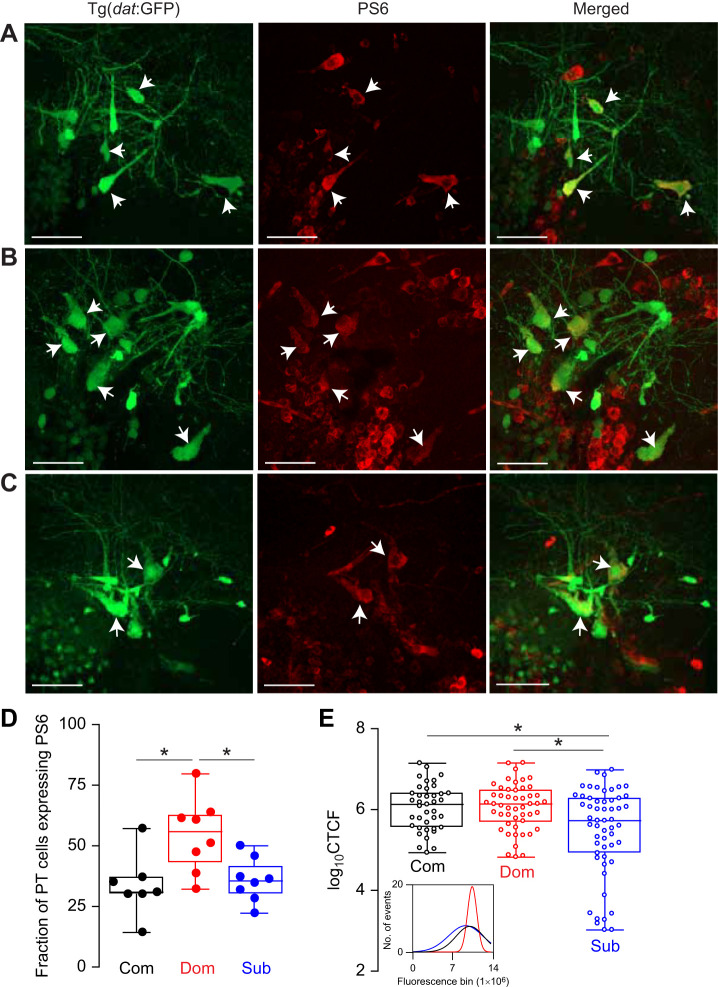
**Effect of social dominance on PS6 expression in the PTar/PTac neural cluster.** Representative confocal projections of the PTar/PTac cluster stained for PS6 in Tg(*dat*:GFP) communal (A), dominant (B) and subordinate (C) zebrafish (scale bars: 30 µm). Arrowheads denote PTar/PTac somata expressing PS6. (D) Box plot of the fraction of PTar/PTac (PT) cells with PS6 expression. (E) Box plots of PS6 fluorescence intensity measurements (CTCF, corrected total cell fluorescence) of all PTar/PTac cells analyzed. Inset illustrates the fluorescence intensity frequency distribution. For box plots in D and E, the box denotes 95% of data, the horizontal line within the box denotes the median, and error bars denote minimum and maximum values. One-way ANOVA with Kruskal–Wallis multiple comparison *post hoc* test (*P<0.05).

Next, we tested the hypothesis that status-dependent morphological differences lead to functional differences in neuronal activity. We rationalized that the PT is a network that must maintain its functional integrity as its morphological architecture adapts to evolving social conditions. We tested this hypothesis by measuring PS6 expression in PTar/PTac neurons. In contrast to immediate early gene markers (e.g. *c-fos* and *erg1*) that detect recent changes in gene expression at the level of transcription, PS6 detects ribosomal proteins that have been phosphorylated in the previous ∼1 h. The increase in phosphorylated ribosomes is tied to increased protein synthesis. Thus, PS6 is a useful marker to measure changes in neural activity (Ruvinsky and Meyuhas, 2006; Maruska et al., 2020; Wayne et al., 2023). We found that PTar/PTac expression of PS6 was significantly higher in dominants than in subordinates and communal controls (one-way ANOVA with Kruskal–Wallis multiple comparison *post hoc* test; *P*=0.0111; communals *n*=5, dominants *n*=8, subordinates *n*=8) (Fig. 5D; Movie 3). Moreover, PS6 fluorescence intensity was significantly lower in subordinates than in dominants and communal fish, but there were no differences between communals and dominants (one-way ANOVA with Kruskal–Wallis multiple comparison *post hoc* test; *P*=0.0147) (Fig. 5E). The results suggest that morphological plasticity induced by social subordination leads to functional differences in PT functional activity pattern.

**Fig. 6.**
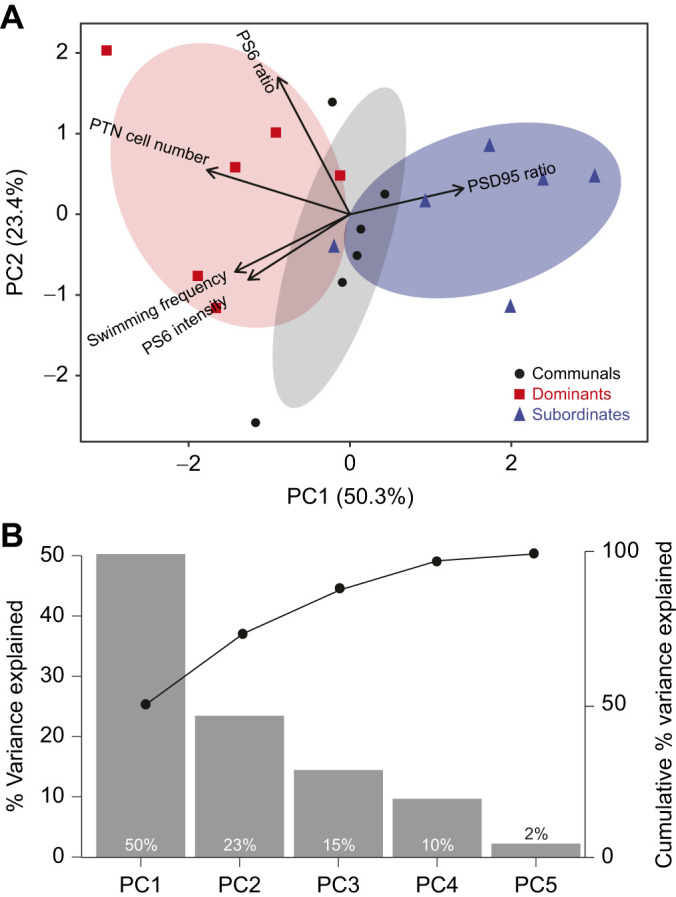
**Ordination based on a principal component analysis (PCA) depicting swimming frequency, posterior tuberculum nucleus (PTN) cell number, and PS6 and PSD-95 expression according to social status on day 14.** (A) The first and second principal components (PC1 and PC2). Points represent samples (6 replicates per social condition), different colors and symbols represent different groups. Ellipses represent 68% confidence intervals of core regions. Loading vectors represent original variables, the directions of arrows represent correlation between the original variable and principal components, and lengths represent the association of original data with principal components. (B) Eigenvalues of the principal components, illustrating the amount of variation explained by each component. The plot is arranged so that the eigenvalues are listed in descending order, from the highest to the lowest. Line plot illustrates the cumulative explained variance.

To examine how physiological and behavioral differences correlate with social status, we conducted a multivariate PCA (Fig. 6). The first two principal components explained about 74% of the variance. The dominant individuals clustered together, where the variables swimming frequency and PTN cell number were negatively correlated and loaded strongly to the PC1 axis. In contrast, the subordinate individuals clustered together, where the variable PSD-95 expression was positively correlated and strongly loaded to the PC1 axis. The communal individuals clustered along PC2, where the variable PS6 intensity was positively correlated and loaded strongly along the PC2 axis. These PCA results confirm the ANOVA analysis highlighting a strong separation between PTN cell number and PSD-95 expression according to social status (Fig. 6). Collectively, the multivariate analysis revealed distinct clusters between dominants and subordinates, emphasizing the divergence in physiological and behavioral activities as animals ascend or descend in social rank.

## DISCUSSION

The PT receives sensory cues from the optic tectum and integrates and relays social information to the spinal cord to modulate motor activity. Here, we examined the effects of social status on the startle escape and swim behaviors and how behavioral adaptations correlate with PTN morphological plasticity. Three principal conclusions can be drawn from this study. Firstly, as social dominance solidifies, the behavior patterns of zebrafish diverge according to social rank. Dominant animals increase their swimming activity and reduce their startle escape sensitivity; subordinates reduce their swimming behavior and increase their startle sensitivity. Secondly, these status-dependent differences correlate with morphological changes in PT cell number, synaptic interconnectivity and activity. Time course experiments suggest that social dominance promotes proliferation in PT cell number, and those differences emerge after 9 days post-dominance formation, while social subordination may lead to neuronal loss. Thirdly, socially driven plasticity of PTN cell number is reversible, suggesting adaptation in motor behaviors and PTN function.

Prior evidence points to possible functional consequences of status-dependent differences in PT morphological architecture on motor activity. Clements and colleagues (2023) have shown that dopaminergic signaling indirectly modulates M-cell excitability by regulating glycinergic inputs to inhibit M-cell excitability. Thus, activation of the intermediate glycinergic inhibitory input leads to an inhibition of startle sensitivity. Interestingly, the authors demonstrated that increased dopaminergic signaling coupled with increased dopamine receptor type 1 (Drd1) expression by glycinergic neurons strengthens the dopaminergic-to-glycinergic pathway to reduce M-cell excitability in dominants. Thus, an increase in PTar/PTac cell number and elevated expression of PS6 activity are likely to increase dopaminergic release to inhibit startle escape in dominants, which is consistent with the current model. In subordinates, having fewer PT cells would reduce dopaminergic signaling, the consequences of which are likely to disinhibit the M-cell and increase startle sensitivity. This is consistent with the reduced expression of PS6 in subordinates, which likely leads to decreased synthesis of dopaminergic signaling-associated proteins (Fig. 5). Further studies examining synaptic transmission between dopaminergic and glycinergic neurons will be necessary to conclusively demonstrate that dopaminergic proliferation leads to increased synaptic communication. Collectively, the results show that the PT is prone to social regulation by undergoing biochemical and morphological plasticity as animals adapt to new social conditions.

The cellular mechanisms underlying the morphological differences in PTar/PTac remain unresolved, but our results hint at two distinct possibilities to guide future studies: neurogenesis during social rise, and cellular loss because of social submission. The adult zebrafish brain continuously regenerates dopaminergic neurons within the diencephalon (Schmidt et al., 2013; Lindsey et al., 2017; Caldwell et al., 2019). Multiple studies inducing lesions within the dopaminergic diencephalic region in amphibians and other vertebrate species have also demonstrated full capability of regeneration (Parish et al., 2007). More interestingly, depending on the specific dopaminergic nuclei, regenerative capacity has been shown to vary, and this is likely to be the result of functional differences (Caldwell et al., 2019). The results also show that there was an initial decline in the number of PTar/PTac cells in both dominants and subordinates compared with communal animals (Fig. 2F, day 7). It is likely that prior social isolation or the initial pairing period of 7 days may have been equally stressful on paired animals. As dominance matures, dominants recover after a week of social interactions, but subordinates continue to experience social stress (Fig. 2F). Indeed, Tea and colleagues (2019) showed that 96 h of social interactions induce social stress and reduces cell proliferation in the telencephalon of subordinate zebrafish compared with dominants, and this reduction was mediated by increased levels of cortisol. Interestingly, cortisol levels in dominants were also elevated relative to those of communally housed fish, with a significant reduction in cell proliferation in the ventral nucleus of the ventral telencephalic area. This suggests that social interactions initially induce stress in the emerging dominant and subordinate fish during the early period of dominance formation, with diverging effects on the rate of cell proliferation. Although direct physiological measurement of stress (e.g. cortisol) was not carried out in our study, hyperventilation and frequent bottom dwelling of subordinates during the 2 weeks of pairing is indicative of anxiety-like and stressful behavior consistent with prior reports (Cachat et al., 2010; Demin et al., 2021; Pancotto et al., 2018). Irrespective of which one of these two scenarios is the leading factor, our results suggest that differences in PT cell number might also be a result of socially induced cellular loss. Indeed, the effect of stress induced experimentally via electric shocks or social manipulation has been extensively documented in many other social animals, with detrimental consequences on brain morphological and functional organization (Ivy et al., 2010; McEwen et al., 2016; Patel et al., 2018). Finally, it is possible that differences in cell number may be due to a shift in cellular identity whereby dopaminergic neurons can downregulate dopamine synthesis and upregulate glutamate (Demarque and Spitzer, 2012). Recent evidence showed that zebrafish PTar/PTac dopaminergic neurons co-express glutamate-synthesizing enzymes (Altbürger et al., 2024). Although we have no evidence to support this view, the apparent increase and decrease in PTar/PTac cell number in dominants and subordinates, respectively, could be due to a shift of dopamine/glutamate expression. However, it remains undetermined whether the PTar/PTac cells possess the cellular capability of shifting their neurotransmitter identity. Coupled with changes in cell number, our results also show status-dependent synaptic plasticity of the PTar/PTac cells. Subordinates had a higher number of PTar/PTac cells expressing PSD-95 compared with dominants and communals (Fig. 4). The functional consequences of increased synaptic formation in subordinates remains unresolved, but it may serve to increase PTar/PTac cellular interconnectivity as a compensatory mechanism to maintain network functionality despite an overall reduction in cell number. Additionally, our results show increased PS6 expression in dominants relative to subordinates, which is consistent with Clements and colleagues' (2023) finding of elevated dopaminergic activity in dominants to regulate M-cell excitability. However, we note that elevated levels of PS6 are not necessarily indicative of higher levels of electrical excitability, but the results suggest that PTN cells are more active biochemically in the synthesis of new proteins that may increase neural excitability.

Our results show that the morphological differences in PT cell number and synaptic density are socially induced and are not due to innate differences. When subordinates were provided with the opportunity for social ascent, they quickly adapted their motor activity to reflect dominant status by reducing their startle sensitivity and increased their swimming activity, which was correlated with a significant increase in PTar/PTac cell number (Fig. 3A–E). However, behavioral adaptation and morphological plasticity in PTar/PTac during social descent were less obvious (Fig. 3B). Dominant animals that lost their social status and became subordinates did not increase their startle sensitivity, nor did they decrease their swimming to subordinate levels as predicted. However, dominants that were forced into submission showed a significant decline in PTar/PTac cell number. This suggests a reluctance to relinquish dominance status, and the pace of behavioral adaptations and morphological plasticity may not be synchronous. The results suggest that brain nuclei involved in motivation and aggression may undergo physiological plasticity at different rates compared with the PTar/PTac. It is likely that the differences in startle sensitivity and swimming frequency would become more pronounced with extended pairing beyond 2 weeks once the new dominance order solidifies.

It is noteworthy that subordinates that remained submissive during the second pairing (having been subordinates for a total of 28 days) displayed depressive-like behavior whereby their swimming activity declined even more than what was observed during the first 2 weeks. Most remarkably, their startle sensitivity began to shift in the opposite direction and they became less sensitive to startle stimuli compared with short-term social subordinates. This phenomenon of learned helplessness has been described extensively as the animals' failure to escape shock induced by uncontrollable aversive events. Seligman and Maier theorized that animals learned that outcomes were independent of their responses, and that this learning undermined efforts to escape (Maier and Seligman, 2016; Seligman and Maier, 1967). A similar occurrence is observed in zebrafish exposed to 3 months of social submission, leading them to display characteristics of learned helplessness with minimal swimming and startle escape response, which is consistent with our current results (F.A.I. and S. Sanders, unpublished observations). Comparison of the startle escape response sensitivity in 14 day subordinates with that of 28 day subordinates shows a significant difference (compare Fig. 1A with Fig. 3B). A 28 day period of social submission appears to cause those animals to reduce their startle sensitivity with a further decline, albeit modest, in swimming frequency compared with that of 14 day social subordinates. The results suggest that extended exposure to social submission beyond 28 days is likely to further amplify and fully manifest the effects of learned helplessness.

Behavioral and cellular plasticity in the startle escape response in other teleost fish species such as cichlids (*Astatotilapia burtoni*) and goldfish (*Carassius auratus*) has also been reported (Medan and Preuss, 2014). As with zebrafish, not only is the M-cell modulated by direct and indirect descending neuromodulatory inputs including serotonin (McLean and Fetcho, 2004; Medan and Preuss, 2014; Pereda et al., 1992; Whitaker et al., 2011) but also its sensitivity is socially regulated, and depends on serotonergic modulation (Whitaker et al., 2021). Interestingly, the response of the M-cell in dominant cichlids is enhanced compared with that of subordinates (Whitaker et al., 2011), suggesting species-dependent adaption to different ecological forces. In cichlids, body coloration is socially regulated whereby dominants are conspicuously brighter than subordinates (Korzan et al., 2008). Thus, despite similarities in brain morphological and functional organization among teleosts, differences in selective pressures in body coloration and predation can alter brain plasticity and decision-making networks to optimize survival as an adaptation to differences in social and ecological environment.

In conclusion, this study demonstrates that social status affects the morphological architecture of the PTN involved in regulating spinal motor circuits. The results show social status-dependent differences in cellular plasticity in dopaminergic cell number, synaptic density and PS6 expression that correlate with adaptions in the startle escape and swim behavior during social rise and fall. Collectively, the findings from this study highlight the impact of social factors in inducing morphological and functional plasticity, the basic principles of which may inform our understanding of other social species with similar structural and functional brain organization.

## Supplementary Material

10.1242/jexbio.248148_sup1 Supplementary information
