# Supplementary material for: Socially induced plasticity of the posterior tuberculum and motor behavior in zebrafish (Danio rerio)
Source: J Exp Biol. 2024 Nov 22;227(22):jeb248148. doi: 10.1242/jeb.248148 (PMC11626077; doi:10.1242/jeb.248148)
Supplement: Supplementary information [file jexbio-227-248148-s1.pdf]

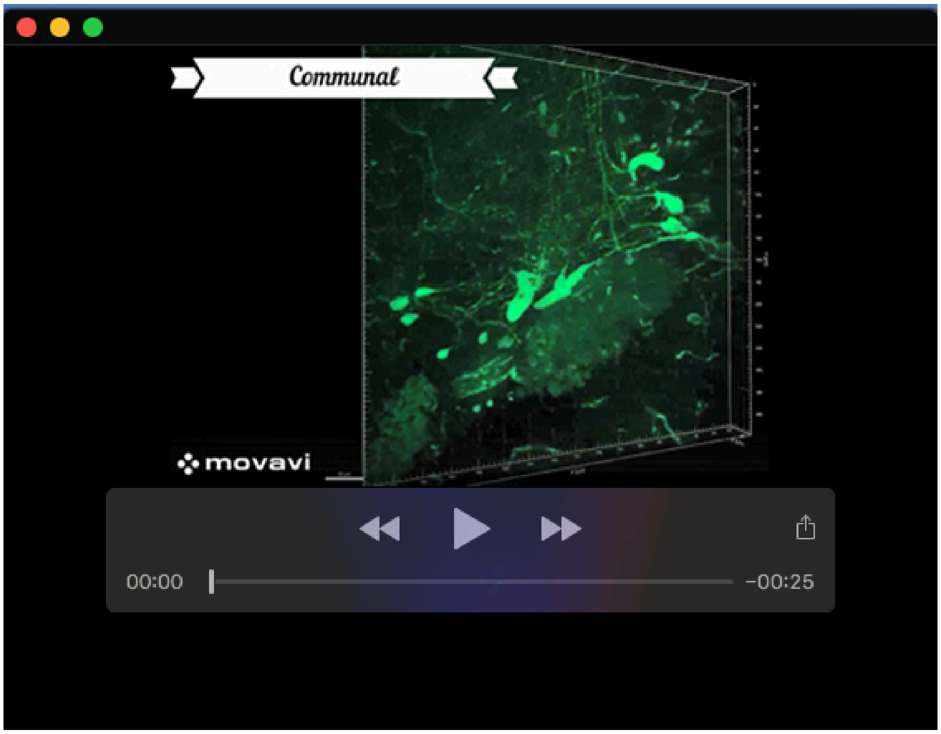

Movie 1. 3D projections of confocal images illustrating the DAT:gfp expressing hypothalamic PTar/PTac and PTp nuclei along with somata digital rendering taken from a communal (clip 1), dominant (clip 2), and subordinate (clip 3) zebrafish. PTar/PTac surface rendering illustrated in red, and PTp somata are illustrated in blue.

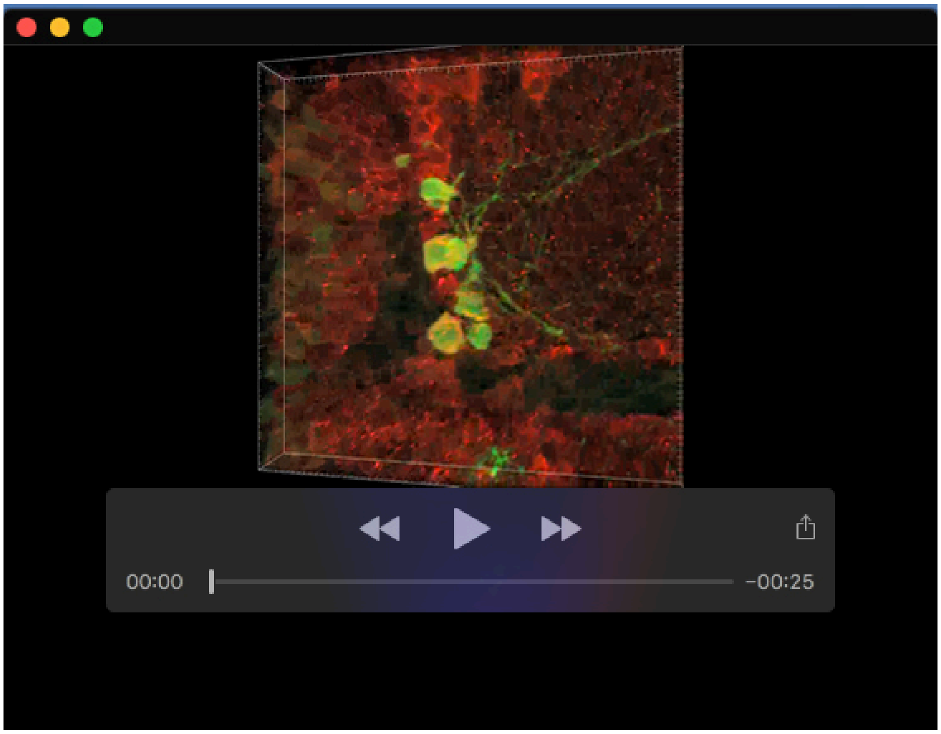

Movie 2. 3D projections of confocal images illustrating the co-expression of PSD-95 (red) in DAT+ PTar/PTac soma (green) taken from a subordinate zebrafish.

Journal of Experimental Biology • Supplementary information

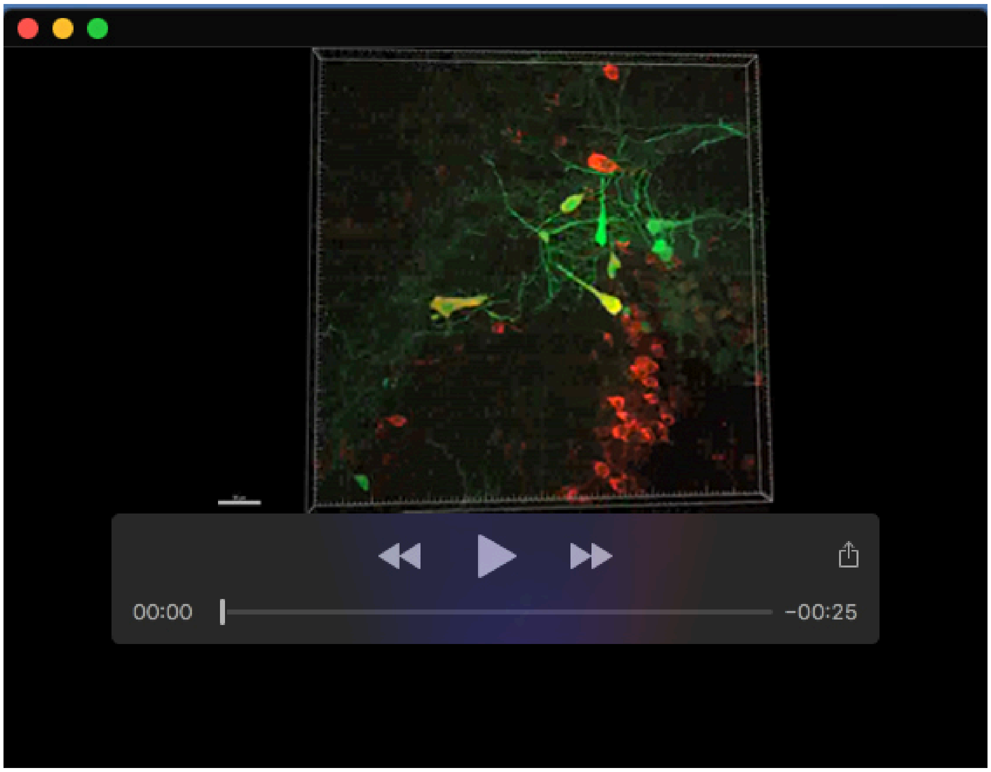

Movie 3. 3D projections of confocal images illustrating the co-expression of PS6 (red) in DAT+ PTar/PTac soma (green) taken from a communal zebrafish. Yellow channel shows the co-localized expressing cells.
